# IL-31-Driven Skin Remodeling Involves Epidermal Cell Proliferation and Thickening That Lead to Impaired Skin-Barrier Function

**DOI:** 10.1371/journal.pone.0161877

**Published:** 2016-08-24

**Authors:** Brijendra Singh, Anil G. Jegga, Kumar S. Shanmukhappa, Ramakrishna Edukulla, Gurjit H. Khurana, Mario Medvedovic, Stacey R. Dillon, Satish K. Madala

**Affiliations:** 1 Division of Pulmonary Medicine, Cincinnati Children’s Hospital Medical Center, Cincinnati, Ohio, United States of America; 2 Division of Biomedical Informatics, Cincinnati Children’s Hospital Medical Center, Cincinnati, Ohio, United States of America; 3 Division of Pathology, Cincinnati Children’s Hospital Medical Center, Cincinnati, Ohio, United States of America; 4 Division of Asthma Research, Cincinnati Children’s Hospital Medical Center, Cincinnati, Ohio, United States of America; 5 Laboratory for Statistical Genomics and Systems Biology, University of Cincinnati, Cincinnati, Ohio, United States of America; 6 Discovery Biology Group, ZymoGenetics, Inc. (a Bristol-Myers Squibb Company), Seattle, Washington, United States of America; Centre National de la Recherche Scientifique, FRANCE

## Abstract

Interleukin-31 (IL-31) is a type 2 helper T-cell-derived cytokine that has recently been shown to cause severe inflammation and tissue remodeling in multiple chronic diseases of the skin and lungs. IL-31 is upregulated in allergic and inflammatory diseases, including atopic dermatitis, asthma, cutaneous T-cell lymphomas, and allergic rhinitis, as well as autoimmune diseases such as systemic erythematosus. Overexpression of IL-31 in T cells causes severe inflammation, with histological features similar to skin lesions of patients with atopic dermatitis. However, the molecular mechanisms involved in IL31-driven pathological remodeling in skin diseases remain largely unknown. Here, we studied the role of IL-31 in skin damage as a result of intradermal administration of recombinant IL-31 into mice. Notably, IL-31 was sufficient to increase epidermal basal-cell proliferation and thickening of the epidermal skin layer. Our findings demonstrate a progressive increase in transepidermal water loss with chronic administration of IL-31 into the skin. Further, analysis of the skin transcriptome indicates a significant increase in the transcripts involved in epidermal-cell proliferation, epidermal thickening, and mechanical integrity. In summary, our findings demonstrate an important role for IL-31 signaling in epidermal cell proliferation and thickening that together may lead to impaired skin-barrier function in pathological remodeling of the skin.

## Introduction

Interleukin-31 (IL-31) is a recently identified T-cell-derived cytokine that is primarily produced by CD4+ T cells polarized toward a Th2 cytokine profile [1]. IL-31 is closely related to the IL-6 family of cytokines and signals through heterodimeric receptors consisting of IL-31 receptor alpha (IL-31RA) and oncostatin M receptor (OSMR) that are expressed constitutively by multiple stromal cells, including epithelial cells and keratinocytes [2], as well as a unique subset of itch-sensitive neurons [3, 4]. Binding of IL-31 to its receptors activates several signaling pathways, including those of Janus kinase-signal transducer and activator of transcriptions or JAK-STAT, mitogen-activated protein kinase, and phosphoinositide-3-kinase or PI3K [5]. IL-31-driven signaling has been shown to regulate a wide range of biological functions, including itch, induction of proinflammatory cytokines, regulation of cell proliferation, and tissue remodeling [3, 6, 7]. Previous studies have shown that IL-31 induces pruritus and severe dermatitis, and also regulates other allergic diseases that are characterized by these skin disorders [1, 8, 9]. Overexpression or administration of recombinant mouse IL-31 (rIL-31) in mice triggered a skin phenotype that in many ways resembled atopic dermatitis (AD) [1]. Moreover, the neutralization of IL-31 signaling has been shown to ameliorate scratching behavior in mouse models of AD [10, 11].

Sustained inflammation and itch are a critical driving force in the initiation and loss of skin-barrier function in AD [12]. The barrier function of the skin is mainly formed by stratum corneum, composed of protein-enriched cells called corneocytes. Impaired barrier function during AD and various other skin diseases results in an increase of water loss from the skin [13, 14]. Transepidermal water loss (TEWL) is a reliable biophysical method that measures the quantity of water that passes from the body to the surrounding atmosphere through the epidermal layer. The existence of the skin barrier is mainly formed by epidermal proliferation and differentiation that begins in the basal layer of the epidermis, which is the deepest layer consisting primarily of proliferating keratinocytes [14]. These cells divide and migrate superficially as they mature. Keratin 14 (K14) is the one of the important proteins expressed by basal dividing keratinocytes of the epidermis [15]. Accumulating evidence indicates that skin damage in AD is associated with T-cell activation, and Th2 T-cell-derived IL-31 appears to be a new link between itchy skin and atopic skin inflammation [1]. A recent study has shown that IL-31RA is expressed in murine neuronal tissue and plays an important role in itch [3]. In support of this, cutaneous injections of IL-31 evoked intense itch and its concentration increased significantly in murine atopic-like dermatitis skin [3]. Moreover, in humans, IL-31 challenge can induce late itch responses and skin erythema [16]. However, the function of IL-31, if any, in skin damage that includes excessive proliferation of epidermal keratinocytes and associated epidermal water loss is largely unknown.

In this study, we found that administration of rIL-31 into the dermis of mouse skin contributes to changes in epidermal morphology and differentiation and skin-barrier disruption. To understand the role of IL-31 in the pathological remodeling of skin, we profiled transcriptional changes that contributed to abrupt proliferation and remodeling in the skin. The data revealed elevated levels for mediators of inflammation, proliferation, barrier function, and extracellular matrix production during IL-31-driven skin damage. Thus, we have identified IL-31 as a critical mediator of pathogenic transcriptional changes and barrier loss in the skin.

## Materials and Methods

### Mice

C57BL/6 mice (Jackson Laboratories, Bar Harbor, ME) at 8–14 weeks of age were used for all of the experiments. Mice were housed in the Cincinnati Children’s Hospital Medical Center (CCHMC) animal facility, which is approved by the American Association for the Accreditation of Laboratory Animal Care. All mice were maintained under aseptic conditions and received sterile food and water. Mice were euthanized by intraperitoneal injection of 200 mg/kg-body weight pentobarbital. Experiments were performed following the Institutional Animal Care and Use Committee (IACUC) regulations, and this study was approved by the CCHMC IACUC.

### IL-31 treatment

Purified rIL-31 protein (20μg/day) (ZymoGenetics, Seattle, WA) was administered to female C57BL/6 mice in 50μl of saline solution via intradermal injection in the center of the shaved back (within a 50-mm radius) daily for 14 days. These doses were selected based upon previous studies [1]. Control mice were given equivalent volumes of saline. TEWL was measured on Days 7 and 14 after rIL-31 administration. After measurement of TEWL, mice were euthanized following standard procedures and skin sections were collected for further analysis.

### Measurement of TEWL

TEWL was measured using the Dermalab instrument DermaLab USB module (Cortex Technology, Hadsund, Denmark), as previously described [17]. TEWL was assessed at Days 7 and 14 on the back of the mice, over the surface where the rIL-31 was injected, and an average of the two readings was used for each mouse.

### Histology

Skin tissues from saline- and rIL-31-treated mice were collected, submerged in 10% neutral-buffered formalin, and embedded in paraffin. Formalin-fixed paraffin-embedded blocks were sliced at 5μm thickness and subjected to hematoxylin/eosin staining. All images were captured using a Leica DM2700 M bright-field microscope (Leica Microsystems, Buffalo Grove, IL), and epidermal thickness was measured using the measured-distance function of MetaMorph imaging software (v6.2; Molecular Devices, Sunnyvale, CA). Quantitative analyses were determined at 10 randomly selected sites from each skin section as the average of the 30 measurements for three sections from each mouse and a total of four mice per group.

### Immunohistochemistry

Formalin-fixed skin sections from saline- and rIL-31-treated mice were prepared and stained with anti-Ki67 antibody (Clone 30–9, Ventana, Tucson, AZ, USA) as detailed in our previous study [18]. The co-immunostained images were captured using a Leica DM2700 M bright-field microscope (Leica Microsystems).

### RNA preparation, whole-transcriptome Shotgun Sequencing (RNA-Seq), and real-time PCR

Total RNA was extracted from skin homogenates using the RNeasy Mini Kit (Qiagen Sciences, Valencia, CA), as described previously [19]. From each experimental group, three skin tissue samples were sequenced using an Illumina HiSeq-1000 Sequencer (Illumina, San Diego, CA), as described previously [20]. To identify differentially expressed genes between the skin of saline- and rIL-31-treated mice at Day 14, we performed statistical analysis using the DESeq Bioconductor package [21], which uses a statistical model based on negative binomial distribution of the read counts. Statistically significant genes were selected based on a *P* value cut-off of 0.05; FDR<0.1 and greater than two-fold changes, resulting in 1,016 genes in total. We performed hierarchical clustering for genes on the log-transformed read counts normalized for different lengths of gene-coding regions (RPKM values) for the saline or rIL-31 groups, and a heat map was generated [22]. The ToppFun application of the ToppGene Suite [23] was utilized to identify the most highly enriched biological processes of the IL-31 gene network. Complete RNA-Seq data are available at a gene-expression omnibus or GEO database (http://www.ncbi.nlm.nih.gov/geo/query/acc.cgi?acc=GSE79403; GEO Accession Number: GSE79403).

For real-time PCR, extracted RNA was converted to cDNA and gene-transcript levels were measured using the CFX384 Touch Real-Time PCR detection system (Bio-Rad, Hercules, CA). Relative gene expression was quantified using SYBR green PCR Master Mix (Applied Biosystems), and gene expression was normalized to hypoxanthine-guanine phosphoribosyltransferase or HPRT. The data were analyzed with StepOnePlusTM software 2.1 (Applied Biosystems), as described by the manufacturer. The mouse primers sequences used in this study are provided in Table 1.

**Table 1:** Mouse primers used.

Primers	Forward (5'-3')	Reverse (5'-3')
*Hprt*	GCCCTTGACTATAATGAGTACTTCAGG	TTCAACTTGCGCTCATCTTAGG
*Saa1*	CCAGGATGAAGCTACTCACCA	TAGGCTCGCCACATGTCC
*S100A8*	TCCTTGCGATGGTGATAAAA	GGCCAGAAGCTCTGCTACTC
*S100A9*	GACACCCTGACACCCTGAG	TGAGGGCTTCATTTCTCTTCTC
*Jup*	ACACCATTCCCCTGTTTGTC	CCACACGCTGGATGTTCTC
*Krt1*	TTTGCCTCCTTCATCGACA	GTTTTGGGTCCGGGTTGT
*Il6*	TCCAGTTGCCTTCTTGGGAC	GTGTAATTAAGCCTCCGACTTG
*Il1β*	TGTAATGAAAGACGGCACACC	TCTTCTTTGGGTATTGCTTGG
*Egr1*	CCTATGAGCACCTGACCACA	TCGTTTGGCTGGGATAACTC
*Fn1*	CGGAGAGAGTGCCCCTACTA	CGATATTGGTGAATCGCAGA
*Mmp2*	GGTGCTCCACCACATACAACT	CCCATGGTAAACAAGGCTTC
*Mmp10*	GAGTCTGGCTCATGCCTACC	TGCAACCAGGAATAAGTTGGT
*Trpv1*	CGAGGATGGGAAGAATAACTC	GGATGATGAAGACAGCCTTGA
*Trpv2*	CCAGCCATTCCCTCATCAAAA	AAGTACCACAGCTGGCCCAGTA
*Trpv4*	TCACCTTCGTGCTCCTGTTG	AGATGTGCTTGCTCTCCTTG
*Trpv6*	ATCCGCCGCTATGCACA	AGTTTTTCTCCTGAATCTTTTTCCA
*Trpc3*	GCCAAGCGACGGAGGAATTA	CAGCACACTGGGGTTCAGTT
*Trpc6*	GCTACTACCCCAGCTTCCGGGG	TGGATGGTTGAGGATTGCCTCCACA

*Hprt*, hypoxanthine-guanine phosphoribosyltransferase; *Saa1*, serum amyloid A1; *S100A8*, S100 calcium-binding protein A8; *S100A9*, S100 calcium-binding protein A9; *Jup*, junction plakoglobin; *Krt1*, keratin 1; *Il6*, interleukin 6; *Il1β*, interleukin 1 beta; *Egr1*, early growth response protein 1; *Fn1*, fibronectin 1; *Mmp2*, matrix metallopeptidase 2; *Mmp10*, matrix metallopeptidase 10; *Trpv1*, *2*, *4*, *6*, transient receptor potential cation channel Subfamily V Members 1, 2, 4, 6; *Trpc3*, *6*, transient receptor potential cation channel Subfamily C Members 3, 6

### Statistics

The data were represented as mean ± SEM. Student’s *t*-test was used to compare between two experimental groups. One-way ANOVA with Tukey’s Multiple Comparison test was used to compare different experimental groups. All data were analyzed using GraphPad Prism (version 6; GraphPad, La Jolla, CA), and a *P* value less than 0.05 was considered statistically significant.

## Results

### Role of IL-31 in epidermal thickness and TEWL

**Fig 1:**
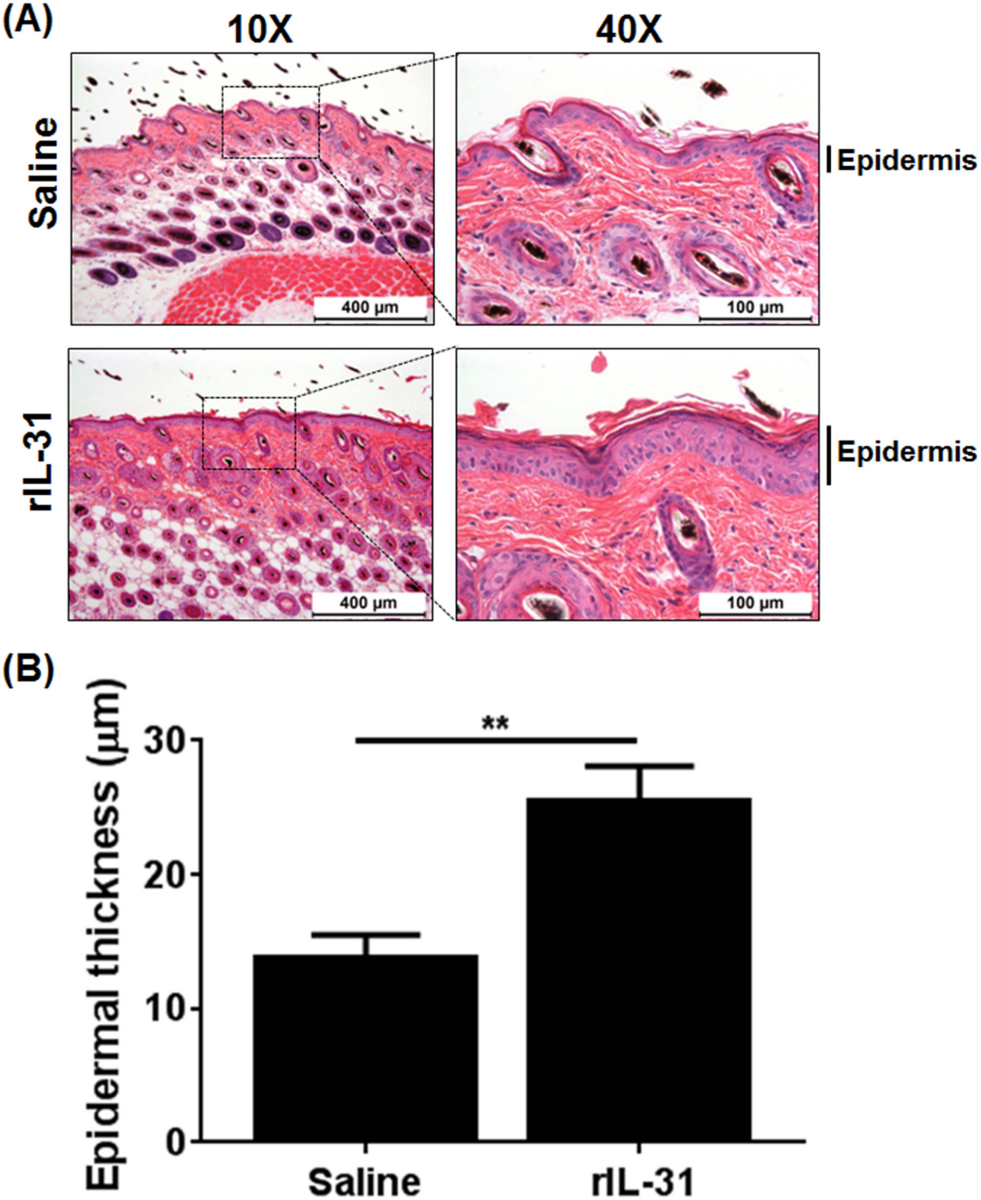
Intradermal administration of IL-31 results in an enhanced epidermal thickness. (**A**) C57BL/6 mice were injected intradermally with saline and rIL-31 (20μg) daily for 14 days and a portion of dorsal skin was excised and processed for thin-sectioning. Haematoxylin/eosin staining was performed to analyze epidermal thickness. (**B**) Quantification of the epidermal thickness was performed using MetaMorph Image analysis software v6.2. Data are cumulative of three independent experiments, with 8–13 total numbers of mice in each group, and represented as mean ± SEM. Unpaired Student’s *t*-test was used to measure significant differences between the groups; **, *P*<0.01.

To study the role of IL-31 in skin damage, IL-31 was administered to C57BL/6 mice via intradermal injections to mimic IL-31 localized in the dermal skin lesions of AD [9, 24–26]. The effect of IL-31 on the epidermal and dermal thickness was assessed by morphometric analysis of hematoxylin/eosin-stained skin sections. Epidermal thickening was significantly increased with IL-31 compared to saline treatment (Fig 1A and 1B). However, administration of rIL-31 had a limited effect on the IL-31-induced increase in dermal thickness (S1 Fig). These data strongly support the role of IL-31 in promotion to induce the pathogenesis of skin damage and epidermal thickness.

**Fig 2:**
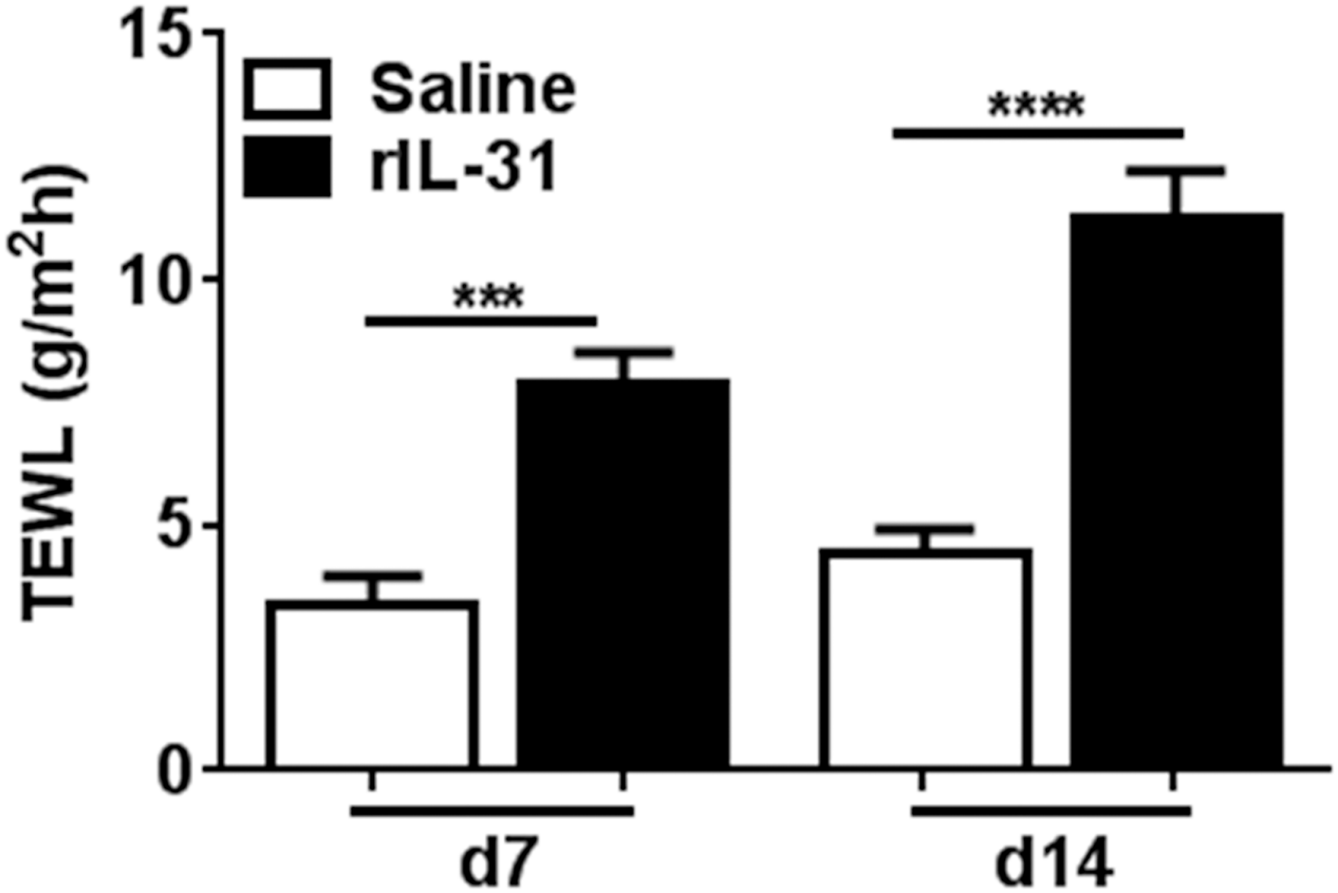
IL-31 increases trans-epidermal water loss (TEWL) in skin. Level of TEWL at Days 7 and 14 on dorsal skin was measured. Data are cumulative of three independent experiments with 8–13 total mice in each group and represented as mean ± SEM. Unpaired Student’s *t*-test was used to measure significant differences between the groups: ***, *P*<0.001; ****, *P*<0.0001.

Disruption of the skin epidermal barrier and an increase in TEWL is a characteristic feature of AD patients [12, 14]. The role of IL-31 in altering skin-barrier function as indicated by TEWL has remained unknown [27]. Therefore, we sought to evaluate the effect of rIL-31 on barrier functions of the skin. We measured TEWL at Days 7 and 14 in mice treated with either saline or rIL-31. TEWL was significantly increased in rIL-31-treated mice as compared to saline-treated mice on Days 7 and 14 (Fig 2), which suggests that IL-31 contributes to disruption of the skin barrier.

### IL-31 increases the proliferation of basal cells in epidermal thickening

**Fig 3:**
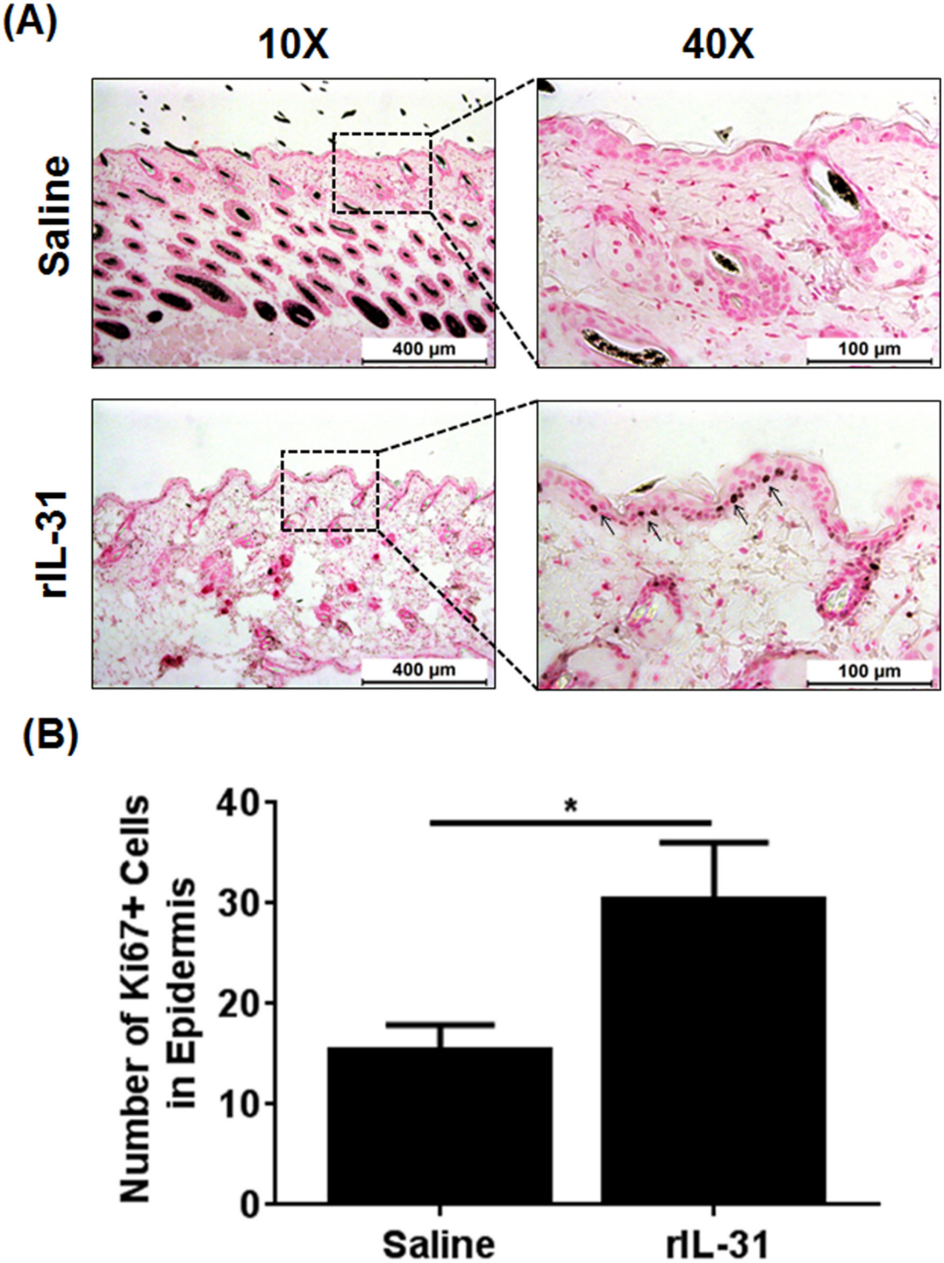
IL-31 increases epidermal cell proliferation in skin. C57BL/6 mice were injected intradermally with saline or rIL-31 (20μg) daily for 14 days. A portion of dorsal skin was excised and processed for thin-sectioning and immunohistochemistry. (**A**) Skin sections from saline- and IL-31-treated mice were immunostained using Ki67 antibody. (**B**) The number of Ki67+ cells in saline- and rIL-31-treated skin were determined using MetaMorph Image analysis software and represented as mean ± SEM. Unpaired Student’s *t*-test was used to measure significant differences between the groups; *, *P*<0.05.

Epidermal differentiation is a complex process by which keratinocytes develop from a proliferative cell type in the epidermis. To understand the role of IL-31 in proliferation and differentiation, we performed immunostainings using anti-Ki67 antibody on saline- and rIL-31-treated skin to visualize the proliferative cells in the epidermis. A significantly increased number of Ki67+ cells were observed in the epidermis of IL-31-treated skin compared to skin treated with saline and were predominantly localized in the basal layer of the epidermis (Fig 3A and 3B). These data suggest that IL-31 induced the proliferation and differentiation of basal cells during progressive skin damage.

### IL-31-dependent signaling pathways

**Fig 4:**
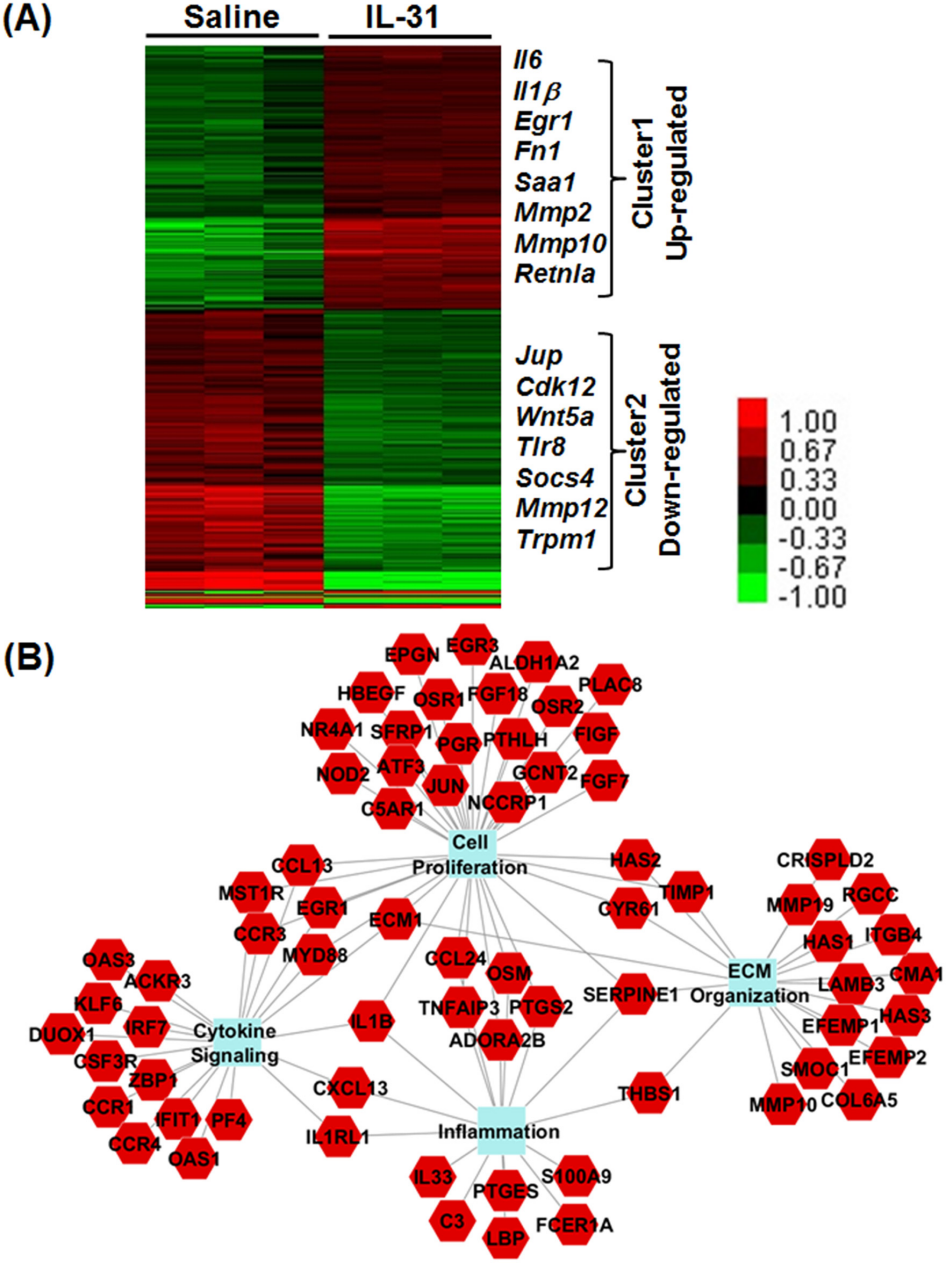
IL-31 regulates expression of genes involved in skin damage. (**A**) C57BL/6 mice were injected intradermally with saline or rIL-31 (20μg) daily for 14 days. A portion of dorsal skin was excised and RNA was isolated. RNA-Seq analysis was performed using next-generation sequencing. Heat map shows two clusters of differentially expressed genes that were either up or down regulated (indicated with color key) in rIL-31-treated mice compared to saline-treated controls. A total of 1,016 significant gene results was analyzed using a *P* value cut-off of 0.05; FDR<0.1 and greater than two-fold changes. (**B**) Network representation of the biological-function enrichment analysis of IL-31-regulated genes identified by RNA-Seq and top-gene function analysis. Solid lines depict the interactions between the genes and biological processes.

To identify differentially expressed genes regulated by IL-31, we performed total-skin transcriptome analysis with RNA isolated from saline- and rIL-31-treated mice. As shown by heat-map analysis, two clusters of differentially expressed genes were observed with rIL-31 treatment compared to saline-treated skin in mice; Cluster 1 and Cluster 2 represent the list of genes upregulated or downregulated, respectively, after IL-31 treatment (Fig 4A). A total of 1,016 significant gene results were analyzed using the *P* value cut-off of 0.05; FDR<0.1 and greater than two-fold changes. IL-31-regulated gene networks identified by the biological function-enrichment analysis included many of those involved in inflammation, proliferation, cytokine-mediated signaling, and tissue remodeling, as highlighted with their gene symbols in the heat-map analysis (Fig 4B).

### IL-31 induces the genes necessary for proliferation and tissue remodeling

**Fig 5:**
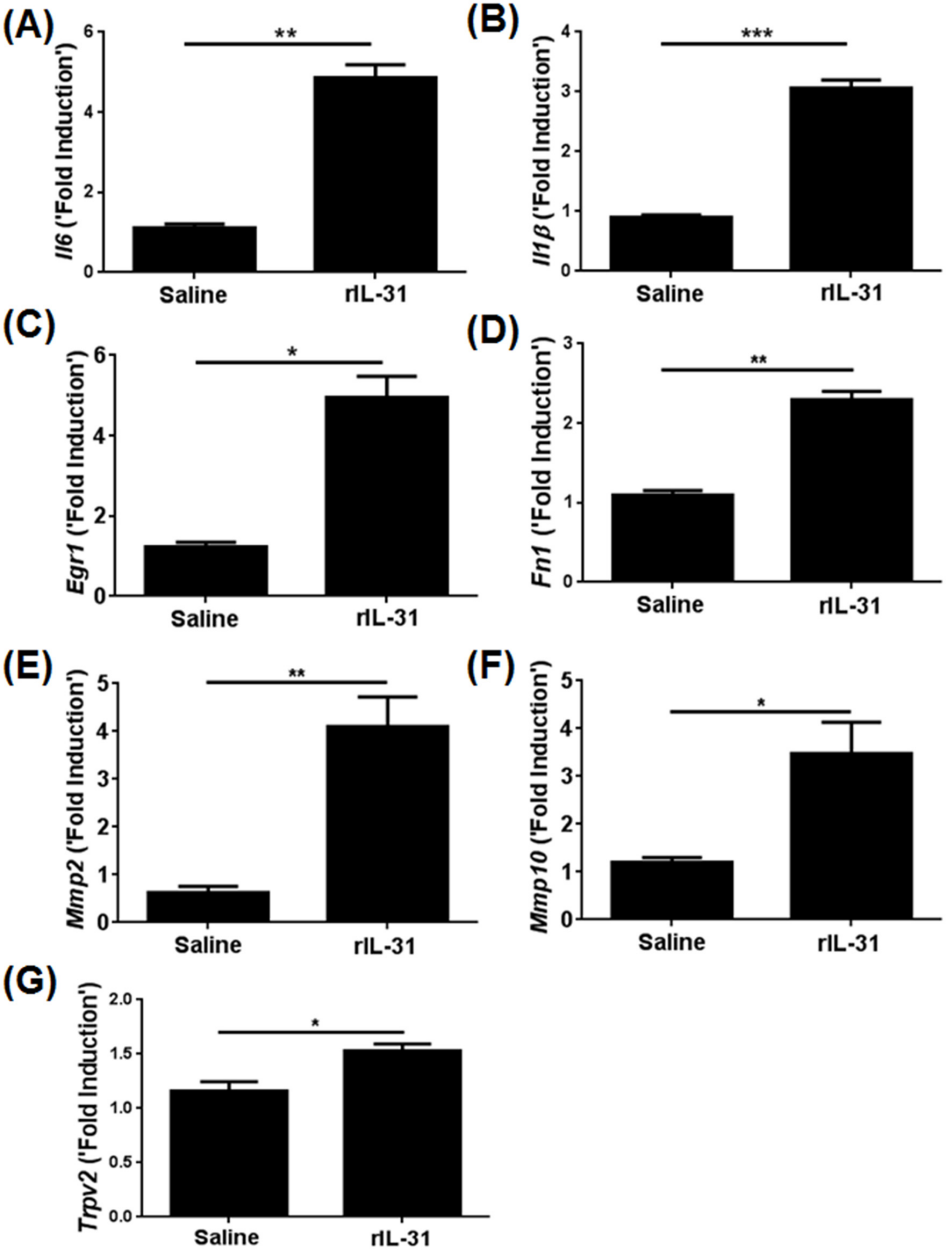
IL-31 increases the expression of genes involved in cell proliferation and tissue remodeling. C57BL/6 mice were injected intradermally with saline or rIL-31 (20μg) daily for 14 days and a portion of dorsal skin was excised. RNA was isolated and converted to c-DNA, and transcripts of genes identified to be regulated through IL-31 network were quantified using RT-PCR. (**A-C**) Proliferative markers—*Il6*, *Il1β*, *Egr1*; Remodelling markers—(**D-F**) *Fn1*, *Mmp2*, *Mmp10*, and (**G**) *Trpv2*. Numbers of mice in each group were 4–5. Data are represented as mean ± SEM, and unpaired Student’s *t*-test was used to measure significant differences between the groups: *, *P*<0.05; **, *P*<0.01; ***, *P*<0.001.

To validate our RNA-Seq data and identify IL-31-induced genes known for proliferation and remodeling in skin, we quantified the transcripts for a number of genes involved in proliferation and tissue remodeling. Among the various genes, *IL-6*, *IL-1β*, *Egr1*, *Fn1*, *Mmp2*, and *Mmp10* transcripts were significantly elevated in the rIL-31-treated skin as compared to saline treatment (Fig 5A–5F). Previous studies have demonstrated that transient receptor potential (TRP) channels are involved in IL-31-mediated itch [3]. To determine whether in vivo rIL-31 directly stimulates sensory neurons inducing itch in skin, we measured the transcripts of different TRP channels known to be involved in itch. Among the various TRP channels, *Trpv2* was found to be significantly increased in the skin of rIL-31-treated mice (Fig 5G). However, other TRP channels (*Trpv1*, *Trpv4*, *Trpv6*, *Trpc3*, and *Trpc6*) were not affected by IL-31 treatment (data not shown).

**Fig 6:**
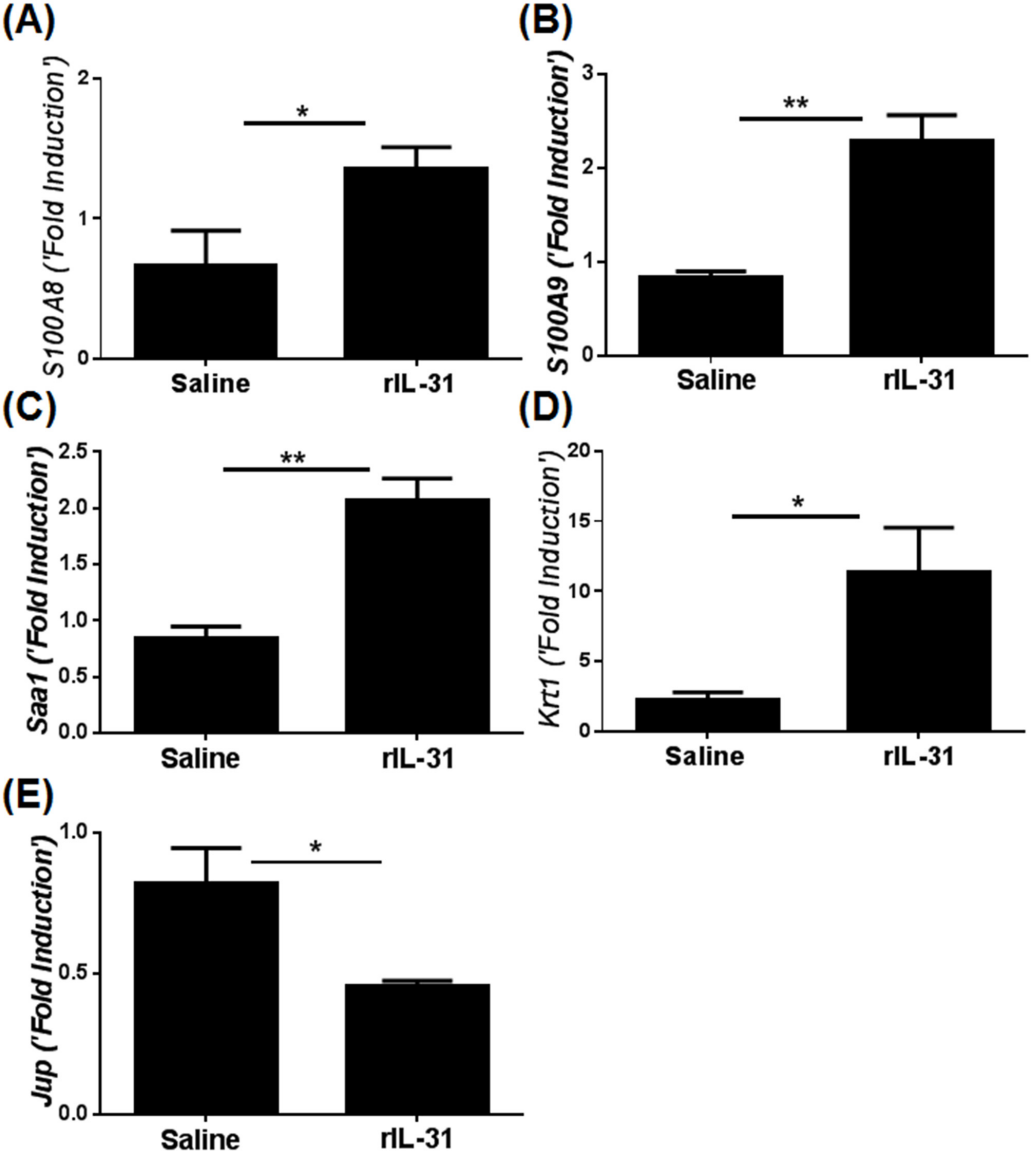
IL-31 increases the expression of genes that alter the mechanical integrity of skin. C57BL/6 mice were injected intradermally with saline or rIL-31 (20μg) daily for 14 days, and a portion of dorsal skin was excised. RNA was isolated and converted to c-DNA. Genes known to be involved in barrier function, inflammation, and mechanical integrity were quantified using qRT-PCR; (**A-B**) *S100A8* and *S100A9*, (**C**) *Saa1*, (**D**) *Krt1*, and (**E**) *Jup*. Numbers of mice in each group were 4–5. Data are represented as mean ± SEM, and unpaired Student’s *t*-test was used to measure significant differences between the groups: *, *P*<0.05; **, *P*<0.01.

Gene transcripts that were implicated in the maintenance of barrier function and mechanical integrity in skin were independently validated using qRT-PCR. Caloprotectin, a heterodimeric complex of *S100A8* and *S100A9*, is known as anti-microbial in human skin and highly expressed during chronic inflammation with compromised barrier function in skin [28, 29]. Both *S100A8* and *S100A9* were significantly increased in skin lesions treated with IL-31 compared to saline (Fig 6A and 6B). Increase in *Saa1* and *Krt1*, which have been shown to augment inflammation and compromise mechanical integrity of skin, were also validated by RT-PCR analysis (Fig 6C and 6D). The loss of desmosomal cadherins such as junction plakoglobin (*Jup*) has been shown to alter keratinocyte proliferation and thickening of the epidermis [30]. Notably, we observed a significant decrease in the transcripts for *Jup* in skin lesions of mice treated with IL-31 compared to saline (Fig 6E).

## Discussion

Impaired skin-barrier function is the most common phenomenon in AD, which indicates the crucial role of epithelial defense in AD pathogenesis [13]. Disturbed skin barrier in patients with AD is related in part to the distribution of the lipid composition of the stratum corneum, which allows penetration of harmful substances into the skin and triggers epidermal proliferation and differentiation [13, 14]. Serum IL-31 level was found to be significantly elevated in AD patients as compared to healthy controls [31, 32]. Moreover, consistent with the human data, the skin phenotype resulting from overexpression or administration of mouse rIL-31 in mice closely mimics that of patients with AD [1]. Therefore, to determine the effects of rIL-31 on skin-barrier function, we measured TEWL. TEWL was significantly increased in rIL-31-treated compared to saline-treated mice, suggesting that IL-31 is involved in disruption of the skin-barrier function. A similar study of AD patients compared to healthy controls, with a two-fold increase in TEWL in non-lesional skin and a four-fold increase in lesional skin, has been reported previously [14]. A study performed in biopsy specimens from 33 AD patients and 13 normal control subjects showed elevated IL-31 transcripts in the AD samples [25]. Moreover, IL-31 expression as detected by immunohistochemistry in inflammatory cells predominates in AD [24]. These data suggest that IL-31 may act as a diagnostic marker for AD patients. Further, to substantiate that epidermal proliferation and differentiation leads to increased TEWL, saline- and rIL-31-treated mouse skin sections were co-immunostained for basal epidermal marker cytokeratin14 and proliferation marker Ki67. In human keratinocytes, the effect of IL-31 on the proliferation of keratinocytes was impaired, as measured by reduced staining for Ki67 [33]. Moreover, IL-31 treatment resulted in a disturbed epidermal differentiation, characterized by abolished K14 expression [33]. However, our new findings in mice with rIL-31 administered intradermally suggest that impaired barrier function in skin mediated by IL-31 involves enhanced proliferation of basal cells in the epidermis. One possible explanation for this is the use of different experimental models and/or dosage of IL-31. In support of our finding, high doses of IL-31 had no effect on filaggrin and desmosomal expression involved in skin differentiation and physical barrier, even though low doses of IL-31 were sufficient to enhance the IL-1 signaling network and several antimicrobial peptides, such as *S100A8* and *S100A9*, involved in inhibiting bacterial growth and skin differentiation [29]. Interestingly, we observed a significant increase in the transcripts for *S100A8*, *S100A9*, *Krt1*, and *Saa1*, which have been shown to maintain skin integrity, barrier function, inflammation, and ichthyosis in human skin [29, 34]. However, we observed no significant differences in filaggrin expression in skin treated with IL-31 (data not shown). Conversely, we observed a significant downregulation of the transcripts for *Jup*, which has been shown to function as a critical regulator of skin-barrier function [30]. In particular, the lack of *Jup* in keratinocytes results in skin ulceration, thickening of the eipidermis, and inflammation [30].

Our RNA-Seq analysis in saline- and rIL-31-treated skin demonstrated that several cytokines and chemotactic factors were involved in IL-31-dependent pathways, including cell proliferation and extracellular matrix organization (*C3*, *IL-33*, *IL-6*, *IL-1β*, *Osm*, *Ccl24*, *Saa1*, and *S100A9*), supporting the involvement of IL-31 in skin physiology. Further, reliability of the RNA-Seq data was confirmed by quantitative RT-PCR, which indicated several differentially expressed genes regulated by IL-31 and involved in proliferation, inflammation, and mechanical integrity of skin (*IL-6*, *IL-1β*, *Saa1*, and *S100A9*). IL-31 plays a crucial role in the development of AD to regulate infiltration of immune cells, such as polymorphonuclear cells (neutrophils and eosinophils) and mononuclear cells (lymphocytes and macrophages), from blood vessels into the epidermal/subepidermal location [1, 19, 24, 35, 36]. IL-31 signals via the heterodimeric receptor composed of IL-31RA and OSMR that is expressed constitutively in keratinocytes of skin. Our new findings demonstrate that intradermal injection of rIL-31 induced epidermal thickness. In support of this, overexpression of IL-31 by T cells was sufficient to cause thickening of the epidermis and inflammatory infiltrates, further supporting the idea of involvement of IL-31 in the promotion of chemotaxis of inflammatory cells to the site of IL-31 production [1]. Future studies are warranted to identify complex interactions among disease-specific co-factors such as the above genes that may dictate clinical phenotypes and mechanical integrity of skin by IL-31.

## Conclusions

The current studies demonstrate that in vivo IL-31 can increase epidermal thickness and TEWL in the skin. Our findings indicate that IL-31 functions as a positive regulator of basal-cell proliferation involved in skin remodeling. Our data also suggest that IL-31 induces changes in skin pathology, in part by enhanced expression of genes involved in proliferation and tissue remodeling. Therefore, future studies are warranted to understand the mechanism of IL-31-driven signalling in skin remodelling and identify novel therapeutic targets in AD and other allergic and inflammatory diseases.

## Supporting Information

S1 Fig Limited effect of administration of rIL-31 on the increase in dermal thickness induced by IL-31. C57BL/6 mice were injected intradermally with saline as control and recombinant IL-31 (rIL-31; 20 μg per injection) daily, for 14 days. A portion of dorsal skin was excised and fixed in 10% buffered formalin, paraffin-embedded, and used for H&E staining. Dermal thickness was measured using MetaMorph Image analysis software. Data is cumulative of three independent experiments, total numbers of mice in each group were 8–13, and represented as mean ± SEM. An unpaired Student’s *t*-test was used to measure the significant difference between the groups.
(PDF)
