# Correction: IL-31-Driven Skin Remodeling Involves Epidermal Cell Proliferation and Thickening That Lead to Impaired Skin-Barrier Function

**DOI:** 10.1371/journal.pone.0170446

**Published:** 2017-01-23

**Authors:** Brijendra Singh, Anil G. Jegga, Kumar S. Shanmukhappa, Ramakrishna Edukulla, Gurjit Kaur Khurana Hershey, Mario Medvedovic, Stacey R. Dillon, Satish K. Madala

The fifth author’s name is spelled incorrectly. The correct name is: Gurjit Kaur Khurana Hershey. The correct citation is: Singh B, Jegga AG, Shanmukhappa KS, Edukulla R, Khurana Hershey GK, Medvedovic M, et al. (2016) IL-31-Driven Skin Remodeling Involves Epidermal Cell Proliferation and Thickening That Lead to Impaired Skin-Barrier Function. PLoS ONE 11(8): e0161877. doi:10.1371/journal.pone.0161877.
