# Supplementary material for: IL-31-Driven Skin Remodeling Involves Epidermal Cell Proliferation and Thickening That Lead to Impaired Skin-Barrier Function
Source: PLoS One. 2016 Aug 24;11(8):e0161877. doi: 10.1371/journal.pone.0161877 (PMC4996532; doi:10.1371/journal.pone.0161877)

Supporting Information

S1 Fig. Limited effect of administration of rIL-31 on the increase in dermal thickness induced by IL-31. C57BL/6 mice were injected intradermally with saline as control and recombinant IL-31 (rIL-31; 20 µg per injection) daily, for 14 days. A portion of dorsal skin was excised and fixed in 10% buffered formalin, paraffin-embedded, and used for H&E staining. Dermal thickness was measured using MetaMorph Image analysis software. Data is cumulative of three independent experiments, total numbers of mice in each group were 8-13, and represented as mean ± SEM. An unpaired Student's *t*-test was used to measure the significant difference between the groups.

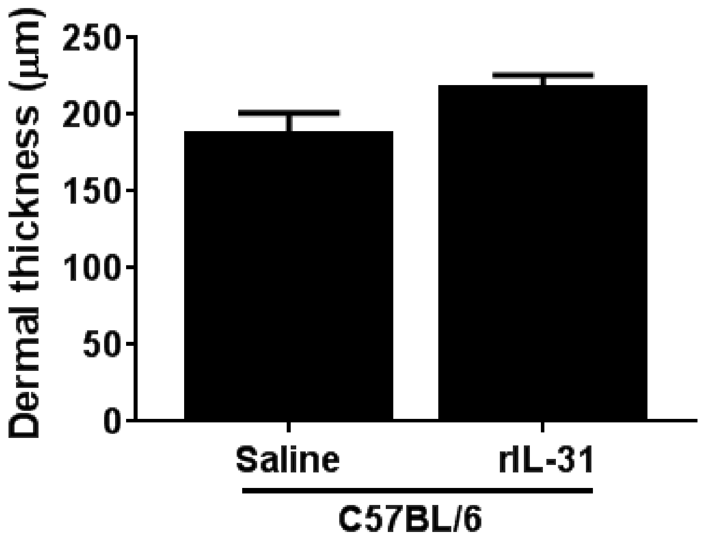

Supplement: S1 Fig — C57BL/6 mice were injected intradermally with saline as control and recombinant IL-31 (rIL-31; 20 μg per injection) daily, for 14 days. A portion of dorsal skin was excised and fixed in 10% buffered formalin, paraffin-embedded, and used for H&E staining. Dermal thickness was measured using MetaMorph Image analysis software. Data is cumulative of three independent experiments, total numbers of mice in each group were 8–13, and represented as mean ± SEM. An unpaired Student’s t-test was used to measure the significant difference between the groups. (PDF) [file pone.0161877.s001.pdf]
